# Supplementary material for: Antibacterial Profile of a Microbicidal Agent Targeting Tyrosine Phosphatases and Redox Thiols, Novel Drug Targets
Source: Antibiotics (Basel). 2021 Oct 27;10(11):1310. doi: 10.3390/antibiotics10111310 (PMC8615086; doi:10.3390/antibiotics10111310)
Supplement: Supplementary file 1 [file antibiotics-10-01310-s001.zip › antibiotics-1397912-supplementary.pdf]

Supplementary Materials

Antibacterial Profile of a Microbicidal Agent Targeting Tyrosine Phosphatases and Redox Thiols, Novel Drug Targets

Kylie White *, Gina Nicoletti and Hugh Cornell

Supplementary Data 1

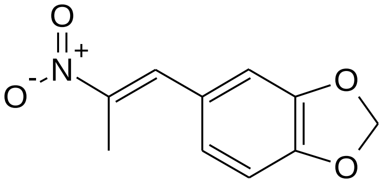

NPBD
(5-(2-nitroprop-1-enyl)-1,3-benzodioxole)

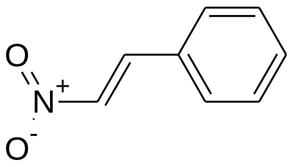

NEB
(2-nitroethenylbenzene)

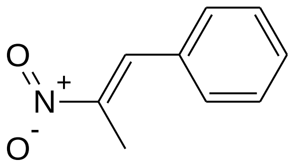

NPB
(2-nitroprop-1-enylbenzene)

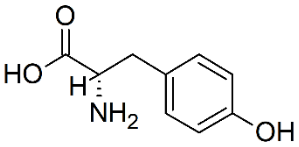

Tyrosine
((2*S*)-2-amino-3-(4-hydroxyphenyl)propanoic acid)

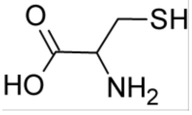

Cysteine
((2*R*)-2-amino-3-sulfanylpropanoic acid)

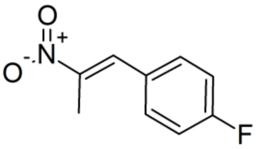

1-fluoro-4-(2-nitroprop-1-enyl]benzene

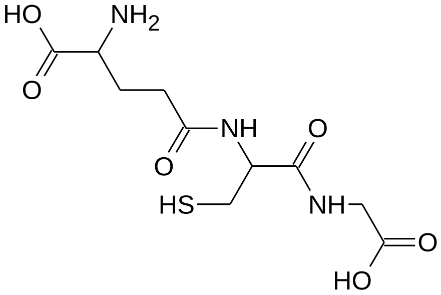

Glutathione
((2S)-2-amino-5-[[(2R)-1-(carboxymethylamino)-1-oxo-3-sulfanylpropan-2-yl]amino]-5-oxopentanoic acid)

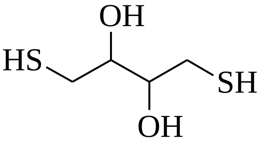

Dithiothreitol
((2*S*,3*S*)-1,4-bis(sulfanyl)butane-2,3-diol)

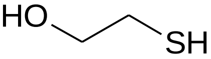

Mercaptoethanol
(2-sulfanylethanol)

Figure S1. Molecular structures of nitroalkene benzenes and interacting molecules.

Supplementary Data 2

Table S1. Physico-chemical characteristics of NPBD (nitropropenyl benzodioxole).

Parameter	Value
IUPAC nomenclature	5-(2-nitroprop-1-enyl)-1,3-benzodioxole
Molecular formula	$C_{10}H_9NO_4$
Optically active	No
Stereoisomers	2
Molecular weight	207.2 g/mol
Appearance	yellow crystalline solid
Melting point	96-98 °C
LogD	3.3
Solubility (DMSO)	300 mg/mL (neutral pH)
Solubility (1:1 DMSO:Ethanol)	150 mg/mL
Solubility (1:1 DMSO: Cremophor EL)	150 mg/mL (with warming)
Solubility (100% Ethanol)	4 mg/mL
Solubility (deionized water)	<12 µg/mL
Heat resistance	200 °C
UV resistance	4-fold loss in activity by MIC at 4 weeks exposure
Stability in solution (DW, DMSO) (dark, 4°C, −20°C)	12 weeks as measured by control MIC assays

Physicochemical studies performed in the Laboratory of Professor Hugh Cornell, RMIT University

Supplementary Data 3—Inhibition of enzymic activity of tyrosine phosphatases

Table S2. NPBD and NPFB inhibition of the enzymic activity of human PTP1B and CD45 and bacterial Yop.

	PTP1B IC_{50}	CD45 IC_{15}	Yop IC_{50}
NPBD	3	3.7	27
NPFB	57	>650	261
Sodium vanadate	0.02	0.06	0.18

NPBD and NPFB were tested at 3-fold dilutions 3.7 to 900 μM and control inhibitor sodium vanadate in 10-fold dilutions 10 nM to 100 μM, against 2mU PTP1B (human), 5 U CD45 (human) and 3 U Yop (bacterial) (Calbiochem) using the ProFluor™ Tyrosine Phosphatase Assay, V1281 (Promega).

NPBD and NPFB (nitropropylene fluorobenzene) show selective and dose-dependent inhibition of test PTPs. NPBD is ~9-fold more active against PTP1B (IC_{50} 3 μM) and ~4.5-fold more active against YOP (IC_{50} 27 μM) than NPFB, IC_{50} 57 and 261 μM respectively. NPBD showed low inhibition of human DSP, CD45. NPFB has broad antimicrobial activity. It is more active than NPBD against LPS-rich gram-negative bacilli but is 4-fold less active against LOS type *Y. enterocolitica* (MIC 64 mg/L) than NPBD (16 mg/L) and ~2-fold less active against representative fungi, mean MIC 9 ±11 mg/L compared to NPBD 4 ±3 mg/L [66].

Supplementary Data 4—Toxicology and Pharmacokinetic studies for NPBD (Compound 1) in rodents.

Part A. Oral absorption and blood levels of Compound (1) in rats after a single dose.

Non-GLP Study conducted at RMIT University Animal Facility Bundoora West (GLP accredited).

Test protocol. Non-GLP study. Sprague-Dawley rats (6 w/o) were administered aqueous suspensions (~100 mL/kg by gavage) of Compound (1) in sterile LPW (100, 500, 1000 and 1250 mg/kg). Blood (100-200 µL) was removed from the tail at 4 and 8 h. Compound (1) was extracted (x2) from serum by toluene and absorbance measured at 370 nm (Hitachi U2000). A spiked control, 100 µg/mL Compound (1) in 50% methanol/water (V/V) and LPW controls were assayed. Animal observations were recorded twice daily for 7 days. Sacrifice and necropsy was performed at 7 days. Gross pathology was recorded and samples of heart, lung, liver, kidney, stomach, spleen, duodenum and colon removed (10% formalin) for histology.

Observations. At doses of 100 and 500 mg/kg there were no adverse symptoms over the 7-day study period and tissues at necropsy looked normal. One rat (1000 mg/kg) was euthanized at 28 h. Postmortem examination revealed spotty and congested lung and bloated stomach. Liver heart and kidney appeared normal but histology was indicative of dilated cardiomyopathy. Remaining 4 rats showed no visible lesions except for some lung congestion. One rat dosed at 1250 mg/kg was distressed and euthanized at 8 h. Lung congestion was noted. Histology showed mild congestion in the liver, lung and myocardium associated with some sinusoidal cell degeneration. Remaining rats had no visible lesions and appeared to tolerate treatment.

Blood levels of Compound (1) were low and dose dependent (Table S3). Oral absorption was ~2.5% for dosing 500 to 1250 mg/kg.

Compound (1) was well tolerated at single doses (NOEL <1000 mg/kg bw) and absorption was ~2.5% for all doses.

Table S3. Serum levels of Compound (1) in the rat following single oral dosing

Compound (1) dose (µg/g)[1]	Sample time h	Blood level Mean (µg/g)
500 fed	4	8
	8	17
500 fasted	4	12
	8	14
1000 fed	4	26
	8	21
1250 fed	4 + 8 h	27

Part B. Acute and 7 day repeat dose toxicity in mice after oral dosing of Compound (1) [NPBD]

**Non-GLP study conducted at RMIT University Animal Facility Bundoora West, GLP accredited animal laboratory.*

Acute dosing: Single doses of Compound (1) in warmed Canola oil at 50, 100 and 200 mg/kg and Canola oil control were administered by oral gavage (vol ≤150 µL) to fasted female BalbC mice (6-8 w, x5 per treatment). Mice were observed for 7 days then sacrificed and necropsied and observed for gross pathology.

7 day repeat dosing: BalbC mice (x5 per group) were dosed daily in the morning for 7 days with a single dose of either Compound (1) at 10, 25 and 100 mg/kg or canola oil alone. animals were observed daily and at 7 days sacrificed and necropsied for gross pathology.

Observations. Compound (1) in canola oil was well tolerated by mice on single oral dosing at 200 mg/kg and no adverse effects were seen on gross pathology at 7 days post dosing. NOEL 200 mg/kg.

NPBD was well tolerated on daily repeat dosing to 7 days at 100 mg/kg and no adverse effects were seen on gross pathology at 7 days. NOEL >100 mg/kg/day.

Part C . Pharmacokinetics of Compound (1) on single oral dose in the rat.

An oral dose of 1000 mg/kg of Compound (1) was well tolerated. The maximum concentration (Cmax) was determined to be 3073 ng/mL at 2 h post-dose. The apparent elimination half-life (Thalf) was 5.85 h. The area under the curve extrapolated to infinite time (AUCinf) was 38628 ng*h/mL with clearance (CL) of 25.9 L/h/kg and volume of distribution (Vd) of 218 L/kg.

Table S4 Pharmacokinetic Parameters of Compound (1)

**Toxicokinetic Parameter**	**Units**	**ICPQN457.A (Oral)**
Dose	mg/kg	1000
Cmax	ng/mL	3073
Tmax	h	2.0
AUC(0-24)	ng*h/mL	36502
Kel	1/h	0.119
Thalf	h	5.85
AUCinf	ng*h/mL	38628
CL	L/h/kg	25.9
Vd	L/kg	218
Number of points for log-linear regression		4
Coefficient of determination		0.9771

Male Sprague-Dawley rats x3 received a single oral dose at 1000mg/kg of Compound (1) in 1% CMC + 0.05% Tween 80. Three blood samples were collected at 0.25, 0.5, 1, 2, 4, 8, 12 & 24 hrs post dose. Compound (1) levels in the plasma were determined by an LC/MS assay. Mean plasma concentrations reported (n=3/group except 24hr where n=6). The LLOQ was 200 ng/mL. Maximum concentration (**Cmax**); time to maximal concentration (**Tmax**); area under the curve to last measurable concentration (**AUCt**); apparent terminal elimination rate (**Kel**); terminal half-life (**Thal**f), calculated as Thalf = Ln(2)/Kel; area under the curve extrapolated to infinite time (**AUCinf**), calculated as AUCinf = AUCt + Clast/Kel where Clast is the last measurable concentration; clearance **(CL),** calculated as CL = Dose / AUCinf; volume of distribution **(Vd)**, calculated as CL/Kel.

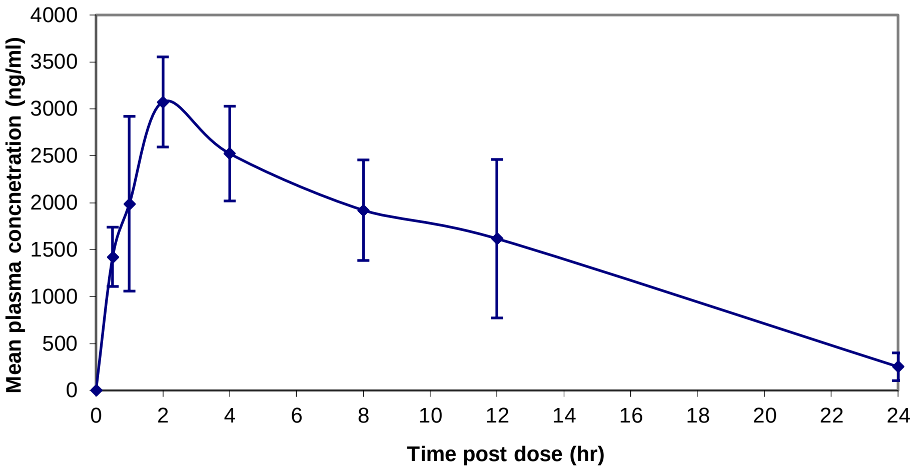

Figure S2. Mean Compound (1) plasma concentrations versus time following a single dose of 1000 mg/kg.

Part D 7 day repeat dose-range finding study in rats*

* GLP study performed in accordance with ICH guidelines.

Protocol: Compound (1) was formulated as a suspension in 1% CMC and 0.05% Tween 80. Rats (5 male, 5 female per treatment) received vehicle or Compound (1) by oral gavage (0, 300, 1100 & 2000 mg/kg), once daily for 7 days. Animals were monitored daily for clinical toxicity, morbidity or mortality. Each animal was subject to a gross necropsy, and a standard tissue list was retained in 10% formalin for possible histopathology. Blood samples for evaluation of serum chemistry, haematology and coagulation parameters were collected from all animals prior to terminal sacrifice on Day 8.

Results summary: Dosing at 1150 mg/kg/day showed 6/10 mortality, 4 animals were found dead and 2 were moribund and sacrificed Mortality at 2000 mg/kg/day was 100%, 9/10 animals being moribund and sacrificed and one found dead on Day 6. All rats on 300 mg/kg survived to sacrifice on Day 8.
Clinical signs of toxicity for 1150 and 2000 mg/kg/day included brown staining around anus, soft faeces, red staining of the cage paper, abdomen and nares, decreased activity, and ruffled haircoat. Moribund sacrificed animals showed clinical signs of toxicity, gross pathological changes on necropsy and changes in haematological and clinical chemistry parameters.
There were some clinical signs in male rats at 300 mg/kg/day, haematological changes in female rats and no changes in clinical chemistry parameters in any animal. Gross necropsy pathology appeared normal but females had increased organ weights
The no-observed adverse-effect level (NOAEL) was <300 mg/kg/day and the lowest lethal dose (LLD) was <1150 mg/kg/day.

Part E. Maximum tolerated dose in nude mice.

Peter McCallum Cancer Centre, Cancer therapeutics Program
Translational Research Laboratory, 305 Grattan St, Melbourne, Vic. Australia
Dr. Carleen Cullinane

Method. Compound 1 (100 mg/mL in DMSO) formulated stock solutions: Vehicle 1: 2-4 mg/mL; 12.5% DMSO, 12.5% ethanol, 12.5% Chremophor EL, 62.5% saline for 1, 5, 10, 15, 20 mg/kg doses. Vehicle 2: 5 mg/mL; 15%DMSO, 12.5% ethanol, 12.5% Chremophor El for 25 mg/kg dose. Stock solutions were formulated by addition of the components in order immediately prior to use.
Groups of two female Balb/c nude mice (Animal Resources Centre, Western Australia) were given Compound 1 (0. 1. 5. 10 15, 20 and 25 mg/kg) at a rate of 0.05 mL/10 g body weight) by intravenous injection twice weekly for three weeks. Mice were weighed daily and monitored for signs of toxicity. Animals were given a feed supplement (Ensure) daily. Blood samples (100 -200 mL from tail) were taken 2 days after Week 3 last dose. Compound 1 was extracted (x2) from serum by toluene and absorbance measured at 370 nm (RMIT).

Results. Mice were temporarily subdued and wobbly for 5-10 min post injections, probably due to osmotic shock. Dosing from 0 to 20 mg/kg using Vehicle 1 when given twice weekly did not adversely affect mouse weight or general health and well-being (Figure 1). The 25 mg/kg dose in Vehicle 2 was also well tolerated (Figure 2). Observed increases in mouse weight is likely to be due to the effects of the feed supplement.
No Maximum Tolerated Dose was established due to the inability to solubilize the compound for IV delivery at concentrations higher than 25 mg/kg mouse weight. Twenty five mg/kg was well tolerated by nude mice after twice weekly dosing for 3 weeks. Blood levels of Compound 1 at 48 h after last dose ranged from <1 μg/mL to <LOD (0.2 μg/mL).

Supplementary Data 5—Inhibition of non-small cell lung cancer tumour cell line *

* Assays were performed by Dr Carleen Cullinane,
Peter McCallum Cancer Centre, Cancer therapeutics Program
Translational Research Laboratory, 305 Grattan St, Melbourne, Vic.

Method. The cytoxicity of NPBD and NPFB (nitropropenyl fluorobenzene) for human non-small cell lung cancer cell line A 549 and human neonatal foreskin fibroblasts (FF) was assayed using the Sulforhodamine B assay [61]. Briefly, cell viability after exposure to drug treatments for 72 h is assessed by the amount of SRB dye bound to cell protein as a proxy for cell mass. Solubilized dye from fixed cells is measured spectrophotometrically by absorbance at 550 nm. The percentage of cell growth is calculated at each drug concentration as: [(Ti-Tz)/(C-Tz)] x 100 for concentrations where Ti ≥ Tz or [(Ti-Tz)/Tz] x 100 for concentrations where Ti < Tz.

Tz = absorbance for time zero growth, Ti is test drug growth and C is growth of FF.

Growth Inhibition (GI_{50}) is reported as the drug concentration that results in a 50% reduction in the net cellular protein increase in control cells at 72 h.

Results. NPBD showed differential toxicity to human NSCLC A549 cells (mean GI_{50} 1.7 μM) compared to neonatal foreskin fibroblasts (GI_{50} 12.5 μM) (Table S5). NPFB was more toxic than NPBD and showed no selective toxicity to the lung cancer cell line.

Table S5. NPBD selective inhibition of NSCLC cell line A549 using SRB cytotoxicity assay.

Compound	Cell line	GI_{50} (μM)
NPBD	$A549_2$	1.4, 2.1
	FF_3	10, 15
NPFB	A549	2.2, 1.8
	FF	2.1, 1.7, 1.6, 2.1

A549 and FF cells (100 μl) seeded into the wells of two 96-well plates and incubated overnight (37°C, 5% CO_2, 95 % air). Plate 1 was fixed with TCA as a measure of cells present at the time of drug addition. NPBD and NPFB (100 μl of 10 concentrations over a 4-log range) were added to each of 5 wells of plate 2 and plate incubated 72 hr. Cells were fixed with 10% TCA and stained with 0.4 % SRB in 1% acetic acid. Wells were washed in DW then 1% acetic acid to remove unbound dye. Protein-bound dye was solubilized in 10 mM Tris base and the absorbance read at 550 nm using an automatic plate reader [61]. GI_{50} is the concentration required to achieve at 72 h a 50% reduction in the net cellular protein increase in control cells.

Supplementary Data 6

Table S6. Susceptibility of test strains to Ciprofloxacin.

**PHYLUM**[a]		**Ciprofloxacin**			
**Order (Family)**	**Species**	**MIC$_{100}$**[b]	±*SD*	**MBC$_{99.9}$**[b]	±*SD*
**FIRMICUTES/ACTINOBACTERIA (Gram positive)**					
Bacillales (Bacillaceae)					
	Bacillus subtilis ATCC6633 (Low G+C)	0.02	*0.01*	0.03	*0*
	Enterococcus faecalis ATCC 29212*	0.125	*0*	0.125	*0*
	E. faecalis clinical strains (7)	0.16	*0.06*	0.19	*0.06*
Bacillales (Staphylococcaeae)					
	Staphylococcus aureus ATCC 29213*	0.19	*0.06*	0.19	*0.06*
	S. aureus clinical strains (´12)	1.4	*1.3*	2.3	*0.9*
Lactobacillalles (Streptococcceae)					
	S. pyogenes ATCC 19615	0.03	*0*	0.06	*0*
	S. pyogenes clinical strains (´10)	0.02	*0.02*	0.03	*0.02*
Lactobacillalles (Lactobacillaceae)					
	Lactobacillus casei RMIT 190/3	0.03	*0*	0.05	*0*
Corynebacteriales (Corynebacteriaceae)					
	Corynebacterium xerosis RMIT 53/5	0.03	*0.00*	0.04	*0.02*
	**Mean**	**0.2**	***0.4***	**0.3**	***0.2***
**PROTEOBACTERIA (α,β,γ) (Gram negative, non-enteric, lipo-oligosaccharide)**					
Pseudomonadales (Moraxellaceae)					
	Acinetobacter calcoaceticus var. anitratus (γ) RMIT3131	0.25	*0*	0.25	*0*
	Moraxella catarrhalis (γ) RMIT 211/2	0.03	*0*	0.04	*0*
Pasteurellales (Pasteurellaceae)					
	Haemophilus influenzae (γ) ATCC 49247	0.03	*0*	0.03	*0*
	Pasteurella multocida (γ) RMIT 284/1-2	0.01	*0*	0.01	*0*
Neisseriales (Neisseriaceae)					
	Neisseria gonorrhoeae (β) RMIT 240/2	0.14	*0.01*	0.03	*0*
Rhizobiales (Brucellaceae)					
	Brucella abortus (α) RMIT 33/1 48h	0.125	*0*	4	*0*
	**Mean**	**0.10**	***0***	**0.7**	***0.0***
**PROTEOBACERIA (γ,ε) (Gram negative, enteric, lipo-oligosaccharide)**					
Campylobacteriales (Campylobactereaceae)					
	Campylobacter jejuni (δ/ε) ATCC 43446 (0:19)	0.06	*0*	0.09	*0*
	C. jejuni NCTC11168	0.03	*0*	0.25	*0*
	C. jejuni 54/1-2	0.03	*0*	0.25	*0*
	C. jejuni 331	0.06	*0*	0.125	*0*
	C. jejuni 1-6 (6)	0.05	*0.03*	0.09	*0.07*
	C. coli	0.06	*0*	0.125	*0*
	C. laridis	1	*0*	2	*0*
	C. sputorum	0.03	*0*	0.125	*0*
	C. foetus	0.03	*0*	0.25	*0*
	C. hyointestinalis	0.06	*0*	0.25	*0*
Bacteroidales (Bacteriodaceae)					
	Bacteroides fragilis NCTC9343	0.3	*0.13*	0.5	*0*
	**Mean**	**0.2**	***0.04***	**0.4**	***0.02***
**PROTEOBACTERIA (γ) (Gram negative, enteric, lipopolysaccharide)**					
Enterobacterales (Enterobacteriaceae)					
	P. mirabilis clinical strains (6)	0.01	*0*	0.02	*0.01*
	P. vulgaris RMIT 281/3	0.003		0.03	
	Escherichia coli ATCC 27853	0.06		0.06	
	E. coli ATCC 25922*	0.03		0.03	
	E. coli RMIT 1110/1 -5 (5)	0.06		0.06	
	K. oxytoca RMIT 180/4	0.006		0.006	

K. pneumoniae RMIT 180/2-6	0.01	*0.02*	0.35	*0.53*
Salmonella Typhimurium ATCC 700720	0.006		0.006	
**Mean**	**0.02**	***0.01***	**0.1**	***0.3***

[a] Phylogenetic taxonomy and nomenclature aligns with the National Center for Biotechnology Information (NCBI) for organisms in the public sequence databases. [b]MIC_{100} and $MBC_{99.9}$ by CLSI microdilution method [59,60] as appropriate for species from a minimum of 3 independent assays. MIC in accepted range for QC type species (*). Test systems contained 1% v/v DMSO. [c]MBC ≤2048 µg/mL in presence of ≤5% DMSO. 24 h MIC/MBC except for *Campylobacter* spp. and *Bacteroides* spp. which were 48 h. Geomean data for each species reported with average titres calculated for multiple strains of a species.

Supplementary Data 7

Table S7. MIC of NPBD (mg/L) against representative clinical strains.

	No: of strains	MIC (mg/L)	MMC (mg/L)
Staphylococcus aureus	12	5 ± 1.9	≥ 512
Streptococcus pyogenes	6	3.2 ± 1	4.5 ± 1.6
Enterococcus faecalis	7	16 ± 0	>512
Proteus mirabilis	6	64 ± 0	>512
Escherichia coli	6	>512	

Strains were collected from Victorian hospital or pathology laboratories, identity confirmed by biochemical tests and stored on nutrient agar slopes at 4 °C. MIC and MMC were determined by the CLSI standard methods for micro broth dilution susceptibility testing of bacteria. Strains were tested twice or three times. Antibiotic susceptibility of bacteria was determined by the CLSI disk diffusion method using a standard panel of antibiotics suitable for each species. Strains were categorized as susceptible (S) or resistant (R) based on zone diameters.

The MIC of recent clinical isolates, including antibiotic-resistant strains, were not different from those of laboratory strains suggesting no cross resistance to the classes of antibiotics tested (Table 1). Nine of the 12 isolates of *S. aureus* were resistant to methicillin. All except ATCC 29213 were resistant to 3 or more antibiotics used in standard therapies for that species. All the clinical strains of *S. aureus* were 4-fold to 16-fold more resistant to ciprofloxacin than ATCC 29213 and 5/11 also showed resistance to norfloxacin. All strains of *Streptococcus pyogenes* were uniformly susceptible to NPBD and ciprofloxacin. *S. pyogenes* has not developed significant resistance to antibiotics except the sulphonamides and intermediate resistance to tetracycline and Cefaclor. The six *Enterococcus faecalis* isolates were resistant to one or more antibiotics excluding vancomycin and were uniformly susceptible to NPBD and ciprofloxacin.

Table S8. Antibiotic susceptibility of *Staphylococcus aureus* clinical strains

	Broth dilution MIC mg/mL		Disc Diffusion Zone diam (mm)										
Strain	NPBD	Ciprofloxacin	Erythromycin	Oxacillin	Penicillin	Vancomycin	Cephalothin	Gentamicin	Chloramphenicol	Ampicillin	Tetracycline	Sulphamethoxa-zole/Trimethoprim	Norfloxacin
Inoculum			E 15	OX 1	P 10	VA 30	KF 30	CN 10	C 30	AMP 10	TE 30	SXP 25	NOR 10
ATCC 29213													
4.00E+04	4	0.25	S (25)	S (19)	R (16)	S (15)	S (30)	S (18)	S (24)	R (8)	R (11)	R (6)	S (29)
2.10E+04	8	0.25											
6.10E+04	4	0.25											
G3178 (17/1)													
4.80E+04	4	2	R (0)	R (0)	R (9)	S (17)	R (10)	R (0)	S (25)	R (20)	S (27)	S (22)	S (25)
1.00E+05	8	2											
6.20E+04	4	2											
G0903 (17/2)													
4.80E+04	4	2	R (9)	R (0)	R (8)	S (16)	R (8)	R (0)	S (26)	R (12)	S (28)	S (21)	S (26)
1.00E+05	8	2											
6.00E+04	4	2											
G6538 (17/3)													
4.60E+04	4	1	R (0)	R (0)	R (8)	S (16)	R (10)	R (0)	S (26)	R (9)	R (11)	R (6)	R (6)
5.00E+04	8	1											
6.50E+04	4	1											
G8080 (17/4)													
3.50E+04	4	4	R (9)	R (0)	R (7)	S (16)	R (9)	R (0)	S (25)	R (16)	S (22)	S (22)	S (28)
8.00E+04	8	4											
7.10E+04	4	4											
G1795 (17/5)													
4.60E+04	4	4	R (10)	R (0)	R (7)	S (16)	R (10)	R (0)	S (26)	R (17)	S (26)	S (22)	S (26)
5.00E+04	8	4											
7.00E+04	4	4											
G6489 (17/6)													

4.00E+04	4	1	R (0)	R (0)	R (7)	S (16)	R (7)	S (18)	S (25)	R (13)	S (26)	S (22)	S (24)
2.10E+04	8	1											
7.20E+04	4	1											
W206													
4.50E+04	4	1	R (0)	R (6)	R (11)	S (16)	S (22)	S (18)	S (25)	R (10)	R (6)	R (6)	R (6)
1.00E+05	8	1											
6.50E+04	4	1											
W241													
4.80E+04	4	4	R (9)	R (0)	R (8)	S (16)	R (9)	R (0)	S (25)	R (8)	R (6)	R (6)	R (6)
1.00E+05	8	4											
7.00E+04	4	4											
W260													
4.60E+04	4	1	S (25)	R (10)	R (15)	S (16)	S (27)	S (18)	S (25)	R (9)	R (11)	R (6)	S (28)
5.00E+04	8	1											
6.00E+04	4	1											
W261													
3.50E+04	4	1	S (25)	S (16)	R (11)	S (15)	S (28)	S (18)	S (24)	R (9)	R (7)	R (6)	R (6)
8.00E+04	8	1											
7.00E+04	4	1											
W318													
4.60E+04	4	1	S (25)	S (15)	R (12)	S (15)	S (26)	S (18)	S (24)	R (9)	R (6)	R (6)	R (6)
5.00E+04	8	1											
6.20E+04	4	1											
**Geo Mean**	**5.0**	**1.4**											
**SD**	**1.9**	**1.3**											

Table S9. Antibiotic susceptibility of *Enterococcus faecalis* clinical strains

	**Broth dilution MIC mg/mL**		**Disc Diffusion Zone diam (mm)**				
**Strain**	**NPBD**	**Ciprofloxacin**	**Ampicillin**	**Penicillin**	**Vancomy-cin**	**Tetracycline**	**Gentamicin**
**Inoculum**			**AMP 10**	**P 10**	**VA 30**	**TE 30**	**CN120**
ATCC 29212							
6.00E+04	16	0.125	S (29)	S (22)	S (17)	R (13)	S (24)
3.20E+04	16	0.125					
X104							
6.20E+04	16	0.25	S (26)	S (21)	I (16)	S (24)	S (17)
6.50E+04	16	0.125					
MU4347							
8.00E+04	16	0.25	S (30)	S (24)	S (17)	R (10)	S (18)
6.00E+04	16	0.125					
MU4382							
8.50E+04	16	0.25	S (26)	S (19)	S (17)	S (23)	S (17)
5.00E+04	16	0.25					
MU4790							
8.50E+04	16	0.125	S (26)	S (19)	S (17)	R (10)	S (17)
5.50E+04	16	0.125					
U5465							
2.00E+04	16	0.125	S (29)	S (22)	S (19)	R (10)	S (21)
3.20E+04	16	0.125					
U5670							
8.50E+04	16	0.25	S (32)	S (25)	I (16)	R (10)	R (9)
6.00E+04	16	0.125					
**Geo mean**	**16.00**	**0.16**					
**SD**	**0.00**	**0.06**					

Supplementary Data 8

Table S10. Compound 1[1] inhibition of growth of *Mycobacterium tuberculosis* [2]

	MIC (g/L)[a]
NPBD	6.25
Isoniazid	0.02-0.1
Rifampicin	0.01-0.02
Ethambutol	1.0-2.5
Streptomycin	0.5

[1] *NPBD and control drugs were prepared in suitable solvents and log$_2$ dilutions in a synthetic liquid medium (SOTON) with 0.35% agar + 10% horse serum, were inoculated with Mtb H37RV, 5 x 10^7 cells/mL and incubated at 37 ℃ to 14 days. MICs were read as complete inhibition of growth at 10-14 days.[37,62].*

[2] *Standard laboratory strain: M. tuberculosis H37RV (ATCC 25618).*

The standard laboratory strain of *M. tuberculosis* H37RV (ATCC 25618) is typical of the species in growth characteristics, drug susceptibility and biochemical activity. Compound 1(3,4-methylenedioxy-β-methyl-β-nitrostyrene) and control drugs were prepared in suitable solvents and log$_2$ dilutions in a synthetic liquid medium (SOTON) with 0.35% agar + 10% horse serum, were inoculated with Mtb H37RV, 5 × 10^7 cells/mL and incubated at 37 °C to 14 days. MICs were read as complete inhibition of growth at 10-14 days [37,62]

NPBD was able to penetrate the wall of *M. tuberculosis* (Mtb) a pathogen with high intrinsic and acquired resistance to antibiotics [63]. *M. tuberculosis* (Mtb) has one kinase PtkA with cognate LMWPTP-like phosphatases, PtpA, and trispecific PtpB, which are essential virulence factors in macrophages [64,65]. PtpA positively regulates expression of the human and mycobacterial alpha sub-unit of ATP synthase, AtpA. Orthovanadate inhibition of PtpA resulted in down-regulation of AtpA in Mtb, reduced ATP synthesis and reduced growth of Mtb in culture [35]. Mtb is sensitive to high ROS levels and has difficulty in maintaining redox homeostasis [36]. PtpA and the anti-oxidative stress systems of M tb are attractive targets for development of antituberucular drugs.

Supplementary Data 9—Generation of non-stable small colony variants in *S. aureus* exposed to NPBD.

The formation of miniature colonies in *S. aureus* at concentrations above the MIC were noted in MBC titrations and some assays. Variant colonies were induced to emerge by extended exposure to NPBD and investigated for phenotypic characteristics and strain stability or reversibility.

Log_2 dilutions of NPBD (0, 32 or 128 mg/L) in MHB with 1% DMSO, were inoculated with log phase cultures of *S. aureus* in 5 mL MHB, standardized to 10^5-10^6 CFU/mL (McFarland opacity standards) and cultures incubated at 30°C, aerobically. At 0, 24 and 48 h, aliquots of culture were diluted 10-fold, plated onto NA and incubated aerobically for up to 48 h at 30°C. Colonies were observed for size, morphology and pigment production. Typical variant colonies (3-5 per plate) from 24 and 48 h exposures were serially sub-cultured onto NPBD-free nutrient agar to test for phenotypic stability. The assay was repeated twice and plates photographed with a digital camera.

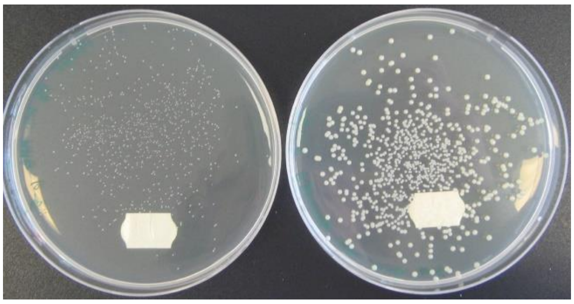

Figure S3. Miniature (left) and normal (right) colonies of *S. aureus* exposed to 32 μg/mL NPBD for 24 h in MHB and 1% DMSO.

Exposure to NPBD 32 & 128 mg/L for 24 h or 48 h produced miniature colonies of <<1 mm diameter (Figure S1). Representative miniature colonies lacked pigment, were Gram positive and of normal microscopic morphology and catalase positive. Subculture of representative miniature colonies to NA in the absence of NPBD gave rise to variably sized colonies at both the first and second subculture (data not shown). Normal colonies had uniform colony diameters (2 mm) and miniature colonies were <<1 mm. Selected miniature colonies from sub-cultured plates all showed the same MIC as control colonies, indicating population reversion to normal parent heterogeneity and no intrinsic population resistance. Atypical colony strains were not further characterized since no stable variant lines were obtained.

Supplementary Data 10 - Binding of nitropropenyl benzodioxole to human serum albumin (HSA)*

**Binding assays were performed in the Laboratory of Prof Hugh Cornell, Department of Chemistry, RMIT University.*

Equilibrium dialysis was carried out with Spectra Por dialysis tubing (molecular weight cut-off 14,000 daltons). Dialysis of a solution of NPBD in PBS containing 15% v/v DMSO in the bag (3 mL) containing 5% HSA, approximately physiological concentration, was carried out for 24 h at room temperature against PBS (200 mL) containing 1% v/v DMSO. A magnetic stirrer was used to circulate the buffer and dialysis bag. All experiments used approximately 3 mg of NPBD in the dialysis bag. At the end of dialysis, the contents of the bag were transferred to a 10 mL volumetric flask and made up to the mark with PBS. Experiments with NPBD in PBS/DMSO but without HSA were performed under the same conditions. Determination of the NPBD concentrations in the bag and in the dialysate was by measurement of absorbance of the solution at 370 nm in a UV/visible spectrophotometer using appropriate dilutions in PBS as necessary. The same experiment was performed with an analogue of NPBD, 1-hydroxy-2-methoxy nitropropenyl benzene (HC1), containing –OH and –OCH3 group instead of the methylene dioxy group. This compound is more water soluble than NPBD and may bind to HSA to a different degree. Calculations based on the amount of agent remaining in the bag as a percentage of the total agent (i.e. content in bag + content in dialysate are an approximate measure of the extent of binding at equilibrium). The small volume of the bag compared with that of the dialysis buffer (1.5% of the latter) leads to a small error at low extent of binding. The differences between duplicate results were in most cases less than 5%. Calculation of antimicrobial agent retained in dialysis bag:

$$\%\ \text{agent retained} = \frac{\text{Absorbance bag contents} \times 10 \times \text{dilution factor}}{\text{Absorbance bag contents} \times 10 \times \text{dilution factor} + \text{absorbance of dialysate} \times 200}$$

NPBD and HC1 bind strongly and reversibly to human serum albumin (Table S11). NPBD was extracted x3 into toluene and quantified by absorbance of NPBD at 370 nm. The use of DMSO as a solvent because of the low solubility of NPBD may underestimate the amount of NPBD bound to protein. Extraction methods used to determine NPBD in plasma may appear lower than expected and the difference in binding between NPBD and HC1 may be greater than measured:

Table S11. NPBD and HC1 bind to human serum albumin (HSA).

	% compound retained in dialysis bag					
	Assay 1	Assay 2	Expt 1	Expt 2	**Mean**	±SEM
NPBD + HSA	63.3	61.3	87.2	81.2	**84.2**	4.2
NPBD	9.3	14.2				
HC1 + HSA	46.5	49.2	78.9	78.5	**78.7**	0.3
HC1*	12.4	13.5				

** HC1 (1-hydroxy-2-methoxy nitropropenyl benzene)*

	% recovery no HSA	% recovery HSA
Extraction 1	76	62.5
Extraction 2	17	28.5
Extraction 3	7	9
	100	100

Supplementary Data 11—Biological availability of NPBD in the presence of plasma proteins

NPBD binds strongly and reversibly to human serum albumin (84 ±4%) (measured by equilibrium dialysis using Spectra/Por tubing (Table S12). There is no standard in vitro method for assessing the effect of protein binding on antimicrobial activity. Free active agent can be estimated by in vitro measurements of antibacterial activity in the presence of protein [66]. Plasma, while a poor growth medium, approximates the protein binding capacity of blood. MHB, provides optimum conditions for bacterial growth and resembles human serum in terms of pH, Na^+, K^+, Cl^- content and osmolality [38].

Reduction in the antibacterial activity of NPBD in the presence of increasing plasma concentrations was measured by determination of the MIC and MBC in MHB. Inhibition of antibacterial action is a predictor for lowered bioavailability in blood and tissues. The free concentration of NPBD in test broths was not determined. A >4-fold decrease in MIC was considered indicative of lower bioavailability due to binding to macromolecules. There is no standard in vitro method for assessing the effect of protein binding on antimicrobial activity.

Increasing plasma levels progressively reduced the bacteriostatic and bactericidal activity for *S. pyogenes* and bacteriostatic activity for *S. aureus* ≥16-fold in 50% plasma. The concentration of free NPBD in the plasma treatments was not measured and cannot be inferred from changes in MIC/MBC titres. The data confirms lowered bioavailability of NPBD in the presence of blood proteins and suggests the dosing required for inhibition of staphylococci and streptococci in blood and tissues would be considerably higher than dosing based on the standard MIC titre. There are no established quantitative relationships between blood protein binding in vitro and plasma binding *in vivo.*

Table S12. Effect of plasma on MIC for NPBD against Gram-positive bacteria in MHB.

	MIC[a] *S. aureus*		MIC[a] *S. pyogenes*		MBC *S. pyogenes*	
%plasma in MHB	24h	48h	24h	48h	24h	48h
0	4	8	4	8	16	16
2.5	8	16	8	16	16	16
5	8	16	8	16	32	32
10	16	32	16	32	128	128
20	32	256	32	64	256	256
50	128	>256	64	128	256	256
100	128	>256	256	>256	>256	>256

[a]*MIC and MBC by macrodilution method for 24 or 48 h. Inoculums 1-1.5 × 10^5 CFU/mL. Bioavailability of NPBD demonstrated by changes in the MIC/MBC for S. aureus ATCC 29213 and S. pyogenes ATCC 19615 in the presence of plasma. MIC and MBC determined by macrodilution and aerobic incubation for 24 h and 48 h at 35°C. Log_2 serial dilutions of NPBD prepared in human plasma or in MHB with added 0, 2.5, 5, 10, 20, 50% plasma. Final inoculum 1-3×10^5 CFU/mL. A decrease in MIC/MBC with increasing concentrations of plasma is an indication of reduction in the level of free NPBD. The level of free NPBD in each medium was not directly measured.*

Supplementary Data 12

Table S13. MIC[a,b] for NPBD against laboratory strains with log_{10} increases in cell density to determine the MIC-cell density relationship.

Inoculum density (CFU/mL)	*S. aureus* ATCC 29213	*B. subtilis* ATCC 6633	*C. xerosis* RMIT 53/5	*E. faecalis* ATCC 29212	*M. catarrhalis* 211/2	*P. vulgaris* RMIT 281/3	*Y. enterocolitica* ATCC 23715	*C. jejumi*
5×10⁴	4	8	8	16	16	32	16	64
[a]5×10⁵	8	8	8	32	16	64	16	128
5×10⁶	16-32	16	16	64	16	64	16	512
5×10⁷	64-128	32	32	128	32	128	32	>512
×MIC	≥×8	×4	×4	×8	×2	×8	×2	>×4

[a]*Density for CLSI microdilution method.*
[b]*The MIC at the population density required for an assay (between 5 ×10⁴ to 5 ×10⁷ CFU/mL) was determined for each assay replicate and designated as MIC_i. The MIC_i was used to evaluate the physiological response in terms of multiples of the standard MIC to enable comparisons across assays.*

Inoculum effect varied with species. MIC increase for a 2× log10 increase in density varied 2× to ≥8×. The greatest increase (≥8) was for species for which NPBD, at the concentrations used, was not bactericidal, *S. aureus*, *E. faecalis* and *P. vulgaris* (MBC >512 µg/mL) and the lowest effect was for *M. catarrhalis* and *Y. enterocolitica* for which it is rapidly bactericidal.

The effect of cell density on the MIC of antibiotics varies with species, bacteriostatic or bactericidal action, and mechanism of action[67–69]. Data for *E. coli*, *Salmonella* spp., *S. aureus* and *S. pneumoniae* suggest mechanism of action is most closely related to the inoculum effect [68].
